# Editors’ Message – September 2023

**DOI:** 10.1016/j.ajpc.2023.100581

**Published:** 2023-08-31

**Authors:** Nathan D. Wong, Erin D. Michos

**Affiliations:** a Division of Cardiology, University of California, Irvine School of Medicine, Irvine, California, USA; b Division of Cardiology, Johns Hopkins University School of Medicine, Baltimore, Maryland, USA

We hope this finds you all well and that you have enjoyed your summer! It was also great to see so many of you at our recent *American Society for Preventive Cardiology (ASPC)* Congress in Arlington, Texas.

As many of you are already aware, the *American Journal of Preventive Cardiology (AJPC)* recently received its first impact factor, a very respectable 4.1. This is undoubtedly due to our highly dedicated team of associate editors and general editorial board members, and of course the many experts in preventive cardiology in the United States (US) and beyond who have served as reviewers and/or authors contributing their fine work to the journal. We are also very grateful to the leadership and staff of the American Society for Preventive Cardiology who have supported the journal from its beginning, as well as significantly helping with its dissemination and exposure. We also greatly appreciate our publisher, beginning with Elsevier editor Dr. Koos Admiral in the development of the *Journal* and more recently Diana Goetz, as well as the many key editorial staff who have worked closely with us. All of you who have contributed countless hours of your time and effort and have believed in the *Journal* from its inception at the beginning of 2020 should be congratulated for helping to make this happen!

But certainly, it will be important to maintain and hopefully improve the impact factor in years to come. This will require the continued dedication of all involved, and of course our many contributors globally who we of course encourage to think of submitting their high quality work to the *Journal*. Moving forward, we hope to consider future special issues – two in progress, one on newer therapies for dyslipidemia and another on women's cardiovascular health, will hopefully be our first such special issues. Another recent initiative is the consideration of joint scientific statements and advisories between the ASPC and other like-minded organizations. One such example is the recent publication of a joint statement on the importance of lipid testing as a performance measure in partnership with the National Lipid Association and its *Journal of Clinical Lipidology.* The further consideration of ASPC clinical practice statements which advance the field of preventive cardiology, which are also of great interest to the *Journal's* readership, remain an important priority of the *Journal.* Finally, the need to further disseminate the *Journal* to our international audiences remains an important priority, and we hope our overseas readers will further consider their fine work for submission to the *Journal.*

Several articles of interest to point out in this issue of the *AJPC* include a short report discussing the topic of very high HDL-C and its potential role in cardiovascular risk assessment, as well as research articles on racial and ethnic differences in NT-pro-BNP levels in US adults, the generalizability of the REDUCE-IT trial in a large diabetes cardiovascular outcomes trial, cardiometabolic predictors of a high risk coronary CT angiography phenotype, and a state-of-the-art review article of the implications of the AHA/ACC/HFSA 2022 Heart Failure Guidelines on the prevention of heart failure.

Now with the *Journal* having a very respectable first impact factor, we hope this will further encourage those you who publish in the preventive cardiology space to consider submitting your fine work for the *Journal*, as well as to share the opportunity to publish in the *Journal* with your colleagues. We of course continue to invite you to contact us regarding any suggestions you may have for improving the *Journal* and continuing to move it forward as the eminent journal in the field.

## Declaration of Competing Interest

The authors declare that they have no known competing financial interests or personal relationships that could have appeared to influence the work reported in this paper.

